# Effects of 16 weeks of pyramidal and polarized training intensity distributions in well‐trained endurance runners

**DOI:** 10.1111/sms.14101

**Published:** 2021-11-25

**Authors:** Luca Filipas, Matteo Bonato, Gabriele Gallo, Roberto Codella

**Affiliations:** ^1^ Department of Biomedical Sciences for Health Università degli Studi di Milano Milan Italy; ^2^ Department of Endocrinology, Nutrition and Metabolic Diseases IRCCS MultiMedica Milan Italy; ^3^ IRCCS Istituto Ortopedico Galeazzi Milan Italy; ^4^ Department of Experimental Medicine Università degli Studi di Genova Genoa Italy; ^5^ Centro Polifunzionale di Scienze Motorie Università degli Studi di Genova Genoa Italy

**Keywords:** polarized training, pyramidal training, running performance, training intensity distribution, training periodization

## Abstract

The aim of this study was to investigate the effects of four different training periodizations, based on two different training intensity distributions during a 16‐week training block in well‐trained endurance runners. Sixty well‐trained male runners were divided into four groups. Each runner completed one of the following 16‐week training interventions: a pyramidal periodization (PYR); a polarized periodization (POL); a pyramidal periodization followed by a polarized periodization (PYR → POL); and a polarized periodization followed by a pyramidal periodization (POL → PYR). The PYR and POL groups trained with a pyramidal or polarized distribution for 16 weeks. To allow for the change in periodization for the PYR → POL and POL → PYR groups, the 16‐week intervention was split into two 8‐week phases, starting with pyramidal or polarized distribution and then switching to the other. The periodization patterns were isolated manipulations of training intensity distribution, while training load was kept constant. Participants were tested pre‐, mid‐ and post‐intervention for body mass, velocity at 2 and 4 mmol·L^−1^ of blood lactate concentration (vBLa2, vBLa4), absolute and relative peak oxygen consumption ($\dot{V}O_{2\text{peak}}$) and 5‐km running time trial performance. There were significant group × time interactions for relative $\dot{V}O_{2\text{peak}}$ (*p* < 0.0001), vBLa2 (*p* < 0.0001) and vBLa4 (*p* < 0.0001) and 5‐km running time trial performance (*p* = 0.0001). Specifically, participants in the PYR → POL group showed the largest improvement in all these variables (~3.0% for relative $\dot{V}O_{2\text{peak}}$, ~1.7% for vBLa2, ~1.5% for vBLa4, ~1.5% for 5‐km running time trial performance). No significant interactions were observed for body mass, absolute $\dot{V}O_{2\text{peak}}$, peak heart rate, lactate peak and rating of perceived exertion. Each intervention effectively improved endurance surrogates and performance in well‐trained endurance runners. However, the change from pyramidal to polarized distribution maximized performance improvements, with relative $\dot{V}O_{2\text{peak}}$ representing the only physiological correlate.

## 1 INTRODUCTION

Endurance coaches, athletes and scientists strive to find the best combination of intensity, duration and frequency of training sessions^1^ to achieve the desired physiological adaptation for athletes and the best performance during main competitions.^2^, ^3^ Manipulating these variables differently over time is traditionally referred to as training periodization.^4^ To improve the understanding of training manipulation and monitoring, different training intensity zones have been described, determined by either physiological factors such as lactate threshold, ventilatory thresholds, percentage of the maximum oxygen uptake, percentage of the maximum heart rate, or subjective factors, such as the goal or rate of perceived exertion of a particular session.^5^

The concept of training intensity distribution (TID) is defined as the amount of time that the athlete spends in different zones of training intensity during exercise.^6^ Usually, TID is calculated by using three training zones: zone 1 (Z1), below the first ventilatory threshold; zone 2 (Z2), between the first and the second ventilatory threshold; zone 3 (Z3), above the second ventilatory threshold.^7^ The intensity distribution known as polarized training is defined as having the highest percentage of time spent in Z1, a smaller, but relatively high percentage in Z3, and only a small portion of training in Z2 (ie, Z1 > Z3 > Z2). On the other hand, pyramidal TID is characterized by accumulating a higher percentage of training time in Z2 and less in Z3, but, as in the case of the polarized model, the highest percentage of training is spent in Z1 (ie, Z1 > Z2 > Z3).^5^, ^8^

Several experimental studies have shown the potential benefits of both polarized and pyramidal TID compared to other TID models for endurance sports.^6^, ^9^, ^10^, ^11^ A recent review on this topic^12^ showed that these two models appear to be the most effective for boosting endurance performance in middle‐ and long‐distance runners. Previous research has identified pyramidal training as the primary TID employed by well‐trained and elite endurance athletes, noting that certain world‐class athletes adopt a polarized training distribution in specific phases of the season.^7^, ^13^, ^14^ There seems to be a pattern across the training season, from a focus on high‐volume, low‐intensity training during the preparation period, to a pyramidal TID during the pre‐competition period, and ending with a polarized TID during the competition phase^15^ in both well‐trained and elite runners.

To our knowledge, there are no studies that have compared, under controlled circumstances, the effects of changing the TID in well‐trained endurance athletes’ periodization,^12^ although this is a common practice among athletes. Therefore, the aim of this study was to investigate the effects of modifying TID throughout a 16‐week periodization in well‐trained endurance runners. Specifically, we sought to compare four different periodization patterns: 16‐week pyramidal (PYR), 16‐week polarized (POL), 8‐week pyramidal followed by 8‐week polarized (PYR → POL), and 8‐week polarized followed by 8‐week pyramidal (POL → PYR). Since it has been shown that training load is crucial for adaptation to endurance training,^16^, ^17^ the periodization patterns employed isolated manipulations of TID while keeping training load constant, thus isolating the effect of TID from the manipulations of training load. Our hypothesis was that, consistent with the training cycles typically used by elite endurance athletes,^7^ switching from pyramidal to polarized TID in the final phase of a training period would result in higher performance improvements compared to maintaining the same distribution (pyramidal or polarized) or switching from polarized to pyramidal TID.

## 2 MATERIALS AND METHODS

### 2.1 Participants

Sixty well‐trained male runners (38 ± 7 years, relative peak oxygen consumption ($\dot{V}O_{2\text{peak}}$): 67 ± 4 ml·kg^−1^·min^−1^) were recruited to the study through local running clubs. Inclusion criteria were as follows: (1) relative $\dot{V}O_{2\text{peak}}$ >60 ml·kg^−1^·min^−1^, (2) training frequency more than five sessions per week, (3) running experience >2 years, (4) regularly competing, and (5) absence of known disease or exercise limitations. The study design and procedures were approved by the local research ethics committee (n° 52/20, attachment 4, 14 May 2020) and followed the ethical principles for medical research involving human participants set by the World Medical Association Declaration of Helsinki. Participants were provided with written instructions outlining the procedures and risks associated with the study and gave informed written consent.

### 2.2 Experimental design

**FIGURE 1:**
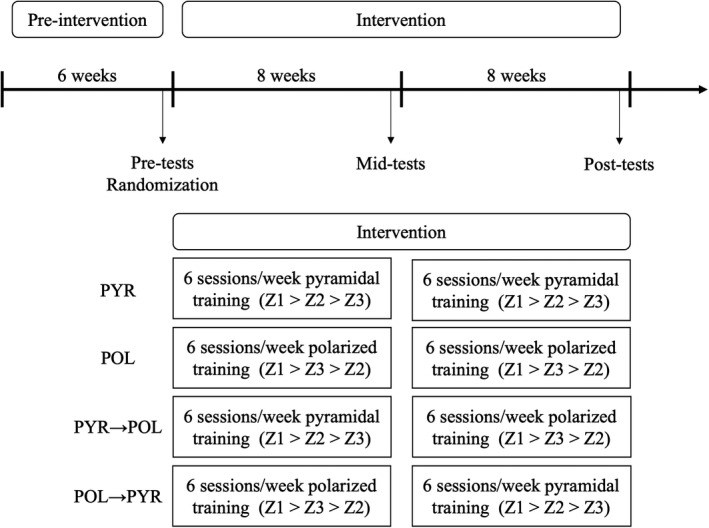
Schematic presentation of the experimental design. Z1, zone 1 (ie, volume below first ventilatory threshold); Z2, zone 2 (ie, volume between first and second ventilatory thresholds); Z3, zone 3 (ie, volume above second ventilatory threshold); PYR, pyramidal training intensity distribution; POL, polarized training intensity distribution; PYR → POL, pyramidal → polarized training intensity distribution; POL → PYR, polarized → pyramidal training intensity distribution

A four‐armed parallel group randomized controlled trial was used. To determine the sample size a priori (software package, G*Power 3.1.9.2), the following input variables were selected as per an *F* test for ANOVA‐repeated measures‐within factors analysis: a statistical power (1 − *β*) of 0.8, a probability α level of 0.05, an effect size *f* of 0.35, four groups and a compliance >90%. These inputs were determined using the literature on training intervention in high‐level endurance athletes. As output variables, an actual power of 0.81 and a critical F of 3.13 were obtained. A sports physician and a certified endurance coach screened the athletes for eligibility. After the pre‐intervention period and the pre‐tests, participants were randomly allocated to one of the four groups based on balanced permutations generated by a web‐based computer program (www.randomization.com) using a 1:1:1:1 ratio. The four groups were matched for age, relative $\dot{V}O_{2\text{peak}}$ and running performance in the 5‐km time trial. A weighting factor was used to properly match the groups at the baseline, dividing athletes in three different blocks based on performance in the pre‐tests. The four groups differed by periodization and/or TID: PYR, POL, PYR → POL and POL → PYR. The member of the research team who conducted the randomization did not take part in the remainder of the study. While participants were aware of their allocation, they were blinded to the true aims of the study. An overview of the experimental protocol is shown in Figure 1.

### 2.3 Pre‐intervention period

Before the intervention, a 6‐week pre‐intervention period was conducted to familiarize subjects with sessions included in the intervention period and with testing protocols. During the pre‐intervention period, participants were instructed to perform only one session in Z2 and one in Z3 each week, combined with a freely chosen volume between 250 and 350 min. They were instructed to complete 6 sessions/week to have a similar training frequency compared to the intervention period. All subjects’ training history during the previous year was monitored using an online training diary (TrainingPeaks, Peaksware LLC), years of running experience, previous peak performance level, and previous/current injuries and diseases. Participants had a mixture of polarized and pyramidal training intensity distribution during the year before the intervention. Pre‐testing was performed at the end of the pre‐intervention (end‐November), and subjects were then randomized into one of four different training groups. No strength and cross‐training were prescribed and performed during the pre‐intervention period. The total volume of training was completed in a running form. All participants were instructed not to change their diet throughout the training period.

### 2.4 Intervention period

#### 2.4.1 Training organization

The training intervention was performed from early December 2019 to the end of March 2020 (16 weeks), which corresponded to the base period for these groups of runners. It consisted of two 8‐week mesocycles structured as 3 + 1 micro‐cycles. Participants were instructed to follow a mesocycle week load structured as follows: weeks 1–3, 5–7, 9–11 and 13–15 had high training loads; weeks 4 and 12 had reduced training loads by 30% compared with the previous three; and weeks 8 and 16 had reduced training loads by 40% compared with the previous three. The three zones model^7^ was used to calculate the TID of the 8‐week mesocycles: zone 1 (Z1), for intensities below first ventilatory threshold; zone 2 (Z2), for intensities between first and second ventilatory thresholds; and zone 3 (Z3), for intensities above second ventilatory threshold. Pyramidal and polarized TID consisted of a higher percentage of training volume spent in Z1 and less in Z2 and Z3, with the proportions of Z2 and Z3 as the main distinguishing characteristic between these two TID (ie, pyramidal: Z1 > Z2 > Z3; polarized: Z1 > Z3 > Z2). The nature of the TID was also verified using the polarization index,^8^ confirming that our distributions were effectively not‐polarized and polarized. Table 1 shows the two different 8‐week training programs. PYR repeated the 8‐week pyramidal mesocycle twice; POL repeated the 8‐week polarized mesocycle twice; PYR → POL completed the 8‐week pyramidal mesocycle and then the polarized one; POL → PYR completed the 8‐week polarized mesocycle and then the pyramidal one. Training impulse (TRIMP) was calculated as volume × intensity according to Lucia and colleagues.^18^ Pyramidal and polarized training distributions were matched using TRIMP for both weekly and mesocycle training loads, to isolate the effect of TID and different periodizations on physiological and performance outcomes.

**TABLE 1:** Plan of the 8‐week mesocycle for PYR and POL. Participants in PYR repeated twice the 8‐week pyramidal mesocycle, in POL repeated twice the 8‐week polarized mesocycle, in PYR → POL completed the 8‐week pyramidal mesocycle and then the polarized one, in POL → PYR completed the 8‐week polarized mesocycle and then the pyramidal one

		Week 1	Week 2	Week 3	Week 4	Week 5	Week 6	Week 7	Week 8
PYR	Mon	70 min Z1	70 min Z1	70 min Z1	50 min Z1	70 min Z1	70 min Z1	70 min Z1	50 min Z1
	Tue	20 min Z1 + 55 min Z2	20 min Z1 + 50 min Z2	20 min Z1 + 55 min Z2	40 min Z1	20 min Z1 + 55 min Z2	20 min Z1 + 50 min Z2	20 min Z1 + 55 min Z2	40 min Z1
	Wed	70 min Z1	70 min Z1	70 min Z1	20 min Z1 + 40 min Z2	70 min Z1	70 min Z1	70 min Z1	Test 1
	Thu	60 min Z1	60 min Z1	60 min Z1	30 min Z1	60 min Z1	60 min Z1	60 min Z1	30 min Z1
	Fri	20 min Z1 + 12 × 2 min Z3 (r. 1 min Z2)	20 min Z1 + 4 × 7 min Z3 (r. 3 min Z2)	20 min Z1 + 3 × 12 × 40 s Z3 (r. 20 s Z2/3 min Z1)	20 min Z1 + 2 × 10 min Z3 (r. 5 min Z2)	20 min Z1 + 12 × 2 min Z3 (r. 1 min Z2)	20 min Z1 + 4 × 7 min Z3 (r. 3 min Z2)	20 min Z1 + 3 × 12 × 40 s Z3 (r. 20 s Z2/3 min Z1)	Test 2
	Sat	Rest	Rest	Rest	Rest	Rest	Rest	Rest	Rest
	Sun	60 min Z1	60 min Z1	60 min Z1	50 min Z1	60 min Z1	60 min Z1	60 min Z1	50 min Z1
	Z1 (min)	300 (77%)	300 (77%)	305 (77%)	210 (78%)	300 (77%)	300 (77%)	305 (77%)	
	Z2 (min)	67 (17%)	62 (16%)	67 (17%)	40 (15%)	67 (17%)	62 (16%)	67 (17%)	
	Z3 (min)	24 (6%)	28 (7%)	24 (6%)	20 (7%)	24 (6%)	28 (7%)	24 (6%)	
	Σ volume (min)	391	390	396	270	391	390	396	
	TL	506	508	511	350	506	508	511	
POL	Mon	70 min Z1	70 min Z1	70 min Z1	50 min Z1	70 min Z1	70 min Z1	70 min Z1	50 min Z1
	Tue	20 min Z1 + 4 × 7 min Z3 (r. 3 min Z2)	20 min Z1 + 8 × 4 min Z3 (r. 2 min Z2)	20 min Z1 + 4 × 7 min Z3 (r. 3 min Z2)	40 min Z1	20 min Z1 + 4 × 7 min Z3 (r. 3 min Z2)	20 min Z1 + 8 × 4 min Z3 (r. 2 min Z2)	20 min Z1 + 4 × 7 min Z3 (r. 3 min Z2)	40 min Z1
	Wed	70 min Z1	70 min Z1	70 min Z1	20 min Z1 + 2 × 10 min Z3 (r. 5 min Z2)	70 min Z1	70 min Z1	70 min Z1	Test 1
	Thu	60 min Z1	60 min Z1	60 min Z1	30 min Z1	60 min Z1	60 min Z1	60 min Z1	30 min Z1
	Fri	20 min Z1 + 12 × 2 min Z3 (r. 1 min Z2)	20 min Z1 + 3 × 12 × 40 s Z3 (r. 20 s Z2/3 min Z1)	20 min Z1 + 12 × 2 min Z3 (r. 1 min Z2)	20 min Z1 + 2 × 10 min Z3 (r. 5 min Z2)	20 min Z1 + 12 × 2 min Z3 (r. 1 min Z2)	20 min Z1 + 3 × 12 × 40 s Z3 (r. 20 s Z2/3 min Z1)	20 min Z1 + 12 × 2 min Z3 (r. 1 min Z2)	Test 2
	Sat	Rest	Rest	Rest	Rest	Rest	Rest	Rest	Rest
	Sun	60 min Z1	60 min Z1	60 min Z1	50 min Z1	60 min Z1	60 min Z1	60 min Z1	50 min Z1
	Z1 (min)	300 (80%)	305 (78%)	300 (80%)	210 (81%)	300 (80%)	305 (78%)	300 (80%)	
	Z2 (min)	24 (6%)	28 (7%)	24 (6%)	10 (4%)	24 (6%)	28 (7%)	24 (6%)	
	Z3 (min)	52 (14%)	56 (15%)	52 (14%)	40 (15%)	52 (14%)	56 (15%)	52 (14%)	
	Σ volume (min)	376	389	376	260	376	389	376	
	TL	504	529	504	350	504	529	504	

Abbreviations: POL → PYR, polarized + pyramidal training intensity distribution; POL, polarized training intensity distribution; PYR → POL, pyramidal + polarized training intensity distribution; PYR, pyramidal training intensity distribution; TL, training load; Z1, zone 1 (ie, volume below first ventilatory threshold); Z2, zone 2 (ie, volume between first and second ventilatory thresholds); Z3, zone 3 (ie, volume above second ventilatory threshold).

#### 2.4.2 Training monitoring

All participants were provided with an online training diary (TrainingPeaks, Peaksware LLC, Lafayette, CO, United States) to record their training. The following variables were registered for each training session: (1) total training duration, (2) total duration in each endurance training zone (time in zone method^19^), and (3) training load calculated using TRIMP.^18^ Individualized heart rate (HR) zones were derived from the incremental ramp test, linking HR zones to ventilatory thresholds. For this purpose, two 5‐min constant load tests were performed at the velocity aligned to the two ventilatory thresholds after the incremental ramp test, and the average HR of the last 30 s was considered as the threshold HR. There were no significant differences among groups in training loads during the intervention period compared with the previous training year. Table 1 shows the arithmetic mean differences among groups for the training variables measured as mean during the 16 weeks.

#### 2.4.3 Pre‐, mid‐ and post‐tests

All participants were asked to stay well‐hydrated, to refrain from consuming alcohol and caffeine for at least 24‐h before testing, and to refrain from engaging in strenuous exercise at least 36‐h prior to testing. They were not allowed to eat during the two hours preceding the tests. All tests were performed under similar environmental conditions (temperature: 6–15 °C; wind: <8 km·h^−1^) on a regular running track and at the same time of day (8:00–10:00) to avoid the influence of circadian rhythm.

The pre‐, mid‐ and post‐tests included the determination of body mass, two incremental tests and a 5‐km time trial. The testing sessions were performed at week 8 and week 16, corresponding to tapering weeks according to the mesocycle structure of the 16‐week training intervention (see Figure 1), to limit the effect of cumulative training fatigue. Tests were performed on two different days, separated by 48 h.

The first testing session was carried out on the Wednesday after 3 days of 40–60 min Z1 sessions. It included a measurement of body mass, a blood lactate profile test and a $\dot{V}O_{2\text{peak}}$ test. The test started without a warm‐up, with 5 min running at 14 km·h^−1^. Running continued and velocity was increased by 0.5 km·h^−1^ every 5 min using an electronic pacesetter. Blood samples were obtained through the ear lobe at the end of each 5‐min bout and were analyzed for whole blood lactate using a portable lactate analyzer (Lactate Pro, Arcray Inc), reported to have good reliability and accuracy.^20^ The smallest detectable change of Lactate Pro for lactate measurement is 1.1%.^20^ The test was terminated when a lactate of 4 mmol·L^−1^ or higher was measured. From this continuous incremental running test, lactate thresholds were calculated as the velocity that corresponded with 2 and 4 mmol·L^−1^ of blood lactate concentration (vBLa2, vBLa4), as recently proposed in similar research.^21^ The blood lactate profile was determined for each runner by plotting lactate vs velocity values obtained during submaximal continuous incremental running. Upon termination of the blood lactate profiling, participants had 20 min of recovery before completing another incremental running test to determine the $\dot{V}O_{2\text{peak}}$. The test was initiated with 1 min of running at 12 km·h^−1^. Running velocity was subsequently increased by 0.5 km·h^−1^ every minute until exhaustion. $\dot{V}O_{2\text{peak}}$ was calculated as the average 30‐s $\dot{V}O_{2}$ measurements. HR >95% of known maximal HR, respiratory exchange ratio >1.05, and lactate >8.0 mmol·L^−1^ were used as criteria to evaluate whether $\dot{V}O_{2\text{peak}}$ was obtained. HR was measured using a Garmin HRM‐Run chest strap (Garmin). $\dot{V}O_{2}$ was measured breath‐by‐breath by a wearable metabolic system (K5, COSMED), reported to have an accurate to acceptable reliability at all metabolic rates.^22^, ^23^ The turbine was calibrated with a 3‐L syringe (M9474, Medikro Oy). Gas analyzers were calibrated with ambient air and gas mixture (16.0% O_2_ and 5.0% CO_2_). The smallest detectable change of K5 for $\dot{V}O_{2}$ measurement at high intensities is 3.4%.^22^

The second testing session was carried out on Friday, 48 h after the first session, and it was preceded by a 30‐min Z1 session. It consisted of a 5‐km time trial on the track. The smallest detectable change of a 5‐km time trial in a competitive environment is 3.2%.^24^ All the athletes were familiar with this distance, as they performed it several times during training and competitions. The test started with their traditional warm‐up routine, standardized within individuals during pre‐, mid‐ and post‐tests. Runners were instructed to perform the test to obtain their best performance over the distance. Researchers provided standardized encouragements at the end of each 400‐m lap. Peak HR (HR_peak_) was calculated using the chest strap as the mean HR during the last 30 s of the time trial. Peak blood lactate (${\text{La}}_{\text{peak}}^{‐}$) was obtained through the ear lobe within 1 min of completion of the time trial using the portable lactate analyzer. Rating of perceived exertion (RPE) was recorded using Borg's 6–20 scale^25^ for each athlete 15–30 min after the end of the time trial. All participants were familiarized with the RPE scale prior to the commencement of the study.

### 2.5 Statistical analysis

All data are presented as arithmetic mean ± standard deviation. Normal distribution and sphericity of data were checked with Shapiro‐Wilk and Mauchly's tests, respectively. Greenhouse‐Geisser correction to the degrees of freedom was applied when assumption of sphericity was violated. To test for differences between groups at pre‐tests among all the physiological and performance variables and in training load, one‐way repeated measures analysis of variance (ANOVA) with Tukey post‐hoc tests was used. Two‐way repeated measures ANOVA (group and time as factors) with Tukey post‐hoc tests were performed to evaluate differences among groups at pre‐, mid‐ and post‐tests for body mass, $\dot{V}O_{2\text{peak}}$, vBLa2, vBLa4, 5‐km time trial time, ${\text{La}}_{\text{peak}}^{‐}$, HR_peak_ and RPE. The variables that predicted time trial performance at pre‐, mid‐ and post‐tests and performance enhancement from pre‐ to post‐tests were identified by multiple linear regression analysis. Body mass, relative $\dot{V}O_{2\text{peak}}$, vBLa2, vBLa4 and ${\text{La}}_{\text{peak}}^{‐}$ were used as predictor variables. Significance was set at 0.05 (two‐tailed) for all analyses. Effect sizes for repeated measure ANOVA are reported as partial eta squared ($\eta_{p}^{2}$), using the small (<0.13), medium (0.13–0.25) and large (>0.25) interpretation for effect size,^26^ while effect sizes for pairwise comparison were calculated using Cohen's *d* and are considered to be either trivial (<0.20), small (0.21–0.60), moderate (0.61–1.20), large (1.21–2.00), or very large (>2.00).^27^ Data analysis was conducted using the Statistical Package for the Social Sciences, version 25 (SPSS Inc.).

## 3 RESULTS

### 3.1 Dropout from the intervention

Four participants (one for each group) were considered as dropouts and were excluded from the final analysis due to their absence from post‐testing and/or <90% adherence to prescribed training sessions. Sixty runners were included in the analysis in total (37 ± 6 years, relative $\dot{V}O_{2\text{peak}}$: 68 ± 4 ml·kg^−1^·min^−1^).

### 3.2 Pre‐test

Table 2 shows that there were no significant differences between PYR, POL, PYR → POL and POL → PYR before the intervention period with respect to all the variables derived from the incremental exercise test to exhaustion.

**TABLE 2:** Baseline variables derived from the incremental exercise test to exhaustion of the participants who completed the 16‐week training intervention, comparing four groups. Values were reported at exhaustion. Data are presented as mean ± standard deviation

	PYR (*n* = 15)	POL (*n* = 15)	PYR → POL (*n* = 15)	POL → PYR (*n* = 15)	*p*
VE (L·min^−1^)	144 ± 8	146 ± 6	145 ± 9	145 ± 8	0.9206
$\dot{V}O_{2\text{peak}}$(L·min^−1^)	4.5 ± 0.3	4.5 ± 0.3	4.5 ± 0.4	4.4 ± 0.3	0.7901
$\dot{V}O_{2\text{peak}}$(ml·kg^−1^·min^−1^)	68 ± 4	69 ± 3	68 ± 5	68 ± 4	0.9538
$\dot{V}{\text{CO}}_{2\text{peak}}$(L·min^−1^)	5.1 ± 0.4	5.2 ± 0.3	5.2 ± 0.4	5.2 ± 0.3	0.8252
RER	1.16 ± 0.03	1.18 ± 0.04	1.16 ± 0.04	1.18 ± 0.02	0.1619
PETO_2_ (mm Hg)	121 ± 5	123 ± 4	123 ± 5	123 ± 4	0.5374
PETCO_2_ (mm Hg)	31 ± 2	30 ± 2	30 ± 1	30 ± 2	0.3355
HR_peak_ (bpm)	183 ± 9	182 ± 8	184 ± 6	182 ± 9	0.8891

Abbreviations: $\dot{V}{\text{CO}}_{2\text{peak}}$, peak carbon dioxide production; $\dot{V}O_{2\text{peak}}$, peak oxygen consumption; HR_peak_, heart rate peak; PETCO_2_, end‐tidal carbon dioxide partial pressure; PETO_2_, end‐tidal oxygen partial pressure; POL → PYR, polarized + pyramidal training intensity distribution; POL, polarized training intensity distribution; PYR → POL, pyramidal + polarized training intensity distribution; PYR, pyramidal training intensity distribution; RER, respiratory exchange ratio; VE, ventilation.

Table 3 shows that there were no significant differences between PYR, POL, PYR → POL and POL → PYR before the intervention period with respect to age, body mass, vBLa2, vBLa4, 5‐km time trial performance, HR_peak_, ${\text{La}}_{\text{peak}}^{‐}$ and RPE.

**TABLE 3:** Baseline characteristics of the participants who completed the 16‐week training intervention, comparing four groups. Data are presented as mean ± standard deviation

	PYR (*n* = 15)	POL (*n* = 15)	PYR → POL (*n* = 15)	POL → PYR (*n* = 15)	*p*
Age (years)	35 ± 6	38 ± 5	38 ± 6	38 ± 6	0.9321
Body mass (kg)	64 ± 3	65 ± 3	65 ± 3	66 ± 3	0.3037
vBLa2 (km·h^−1^)	16.3 ± 1.1	16.4 ± 0.8	16.2 ± 1.2	16.4 ± 1.1	0.9390
vBLa4 (km·h^−1^)	17.3 ± 1.1	17.4 ± 0.8	17.2 ± 1.2	17.4 ± 1.1	0.9572
Time trial time (s)	993 ± 57	998 ± 48	986 ± 56	998 ± 61	0.9433
${\text{La}}_{\text{peak}}^{‐}$ (mmol·L^−1^)	9 ± 2	10 ± 2	10 ± 3	10 ± 2	0.6204
HR_peak_ (bpm)	179 ± 12	177 ± 12	177 ± 12	177 ± 10	0.9598
RPE (6–20)	18 ± 1	18 ± 1	18 ± 1	18 ± 1	0.8951

Abbreviations: ${\text{La}}_{\text{peak}}^{‐}$, peak blood lactate; HR_peak_, heart rate peak; POL → PYR, polarized + pyramidal training intensity distribution; POL, polarized training intensity distribution; PYR → POL, pyramidal + polarized training intensity distribution; PYR, pyramidal training intensity distribution; vBLa2, velocity at 2 mmol·L^−1^ of blood lactate concentration; vBLa4, velocity at 4 mmol·L^−1^ of blood lactate concentration.

### 3.3 Training load

Effective TID and training load of participants in PYR, POL, PYR → POL and POL → PYR are presented in Table 4. No significant differences were calculated between groups for training load.

**TABLE 4:** Average training intensity distribution and training load in PYR, POL, PYR → POL and POL → PYR for week 1–8 and week 9–16. Data are presented as mean ± standard deviation

Group	Week 1–8					Week 9–16					
	Z1 (min)	Z2 (min)	Z3 (min)	Σ volume (min)	TL		Z1 (min)	Z2 (min)	Z3 (min)	Σ volume (min)	TL
PYR	279 ± 40	55 ± 20	25 ± 3	358 ± 63	463 ± 77		279 ± 39	54 ± 19	24 ± 5	358 ± 63	462 ± 78
POL	279 ± 41	21 ± 8	48 ± 9	348 ± 57	464 ± 81		279 ± 40	21 ± 7	48 ± 8	348 ± 56	465 ± 79
PYR → POL	280 ± 37	54 ± 19	24 ± 3	358 ± 63	462 ± 78		279 ± 40	21 ± 9	47 ± 8	347 ± 56	463 ± 77
POL → PYR	279 ± 42	21 ± 7	47 ± 9	348 ± 56	465 ± 80		278 ± 41	55 ± 17	25 ± 3	358 ± 63	464 ± 79
*p*				0.824	0.992					0.812	0.992

Abbreviations: POL → PYR, polarized + pyramidal training intensity distribution; POL, polarized training intensity distribution; PYR → POL, pyramidal + polarized training intensity distribution; PYR, pyramidal training intensity distribution; TL, training load; Z1, zone 1 (ie, volume below first ventilatory threshold); Z2, zone 2 (ie, volume between first and second ventilatory thresholds); Z3, zone 3 (ie, volume above second ventilatory threshold).

### 3.4 Body mass, $\dot{V}O_{2\text{peak}}$ and lactate profiles

**FIGURE 2:**
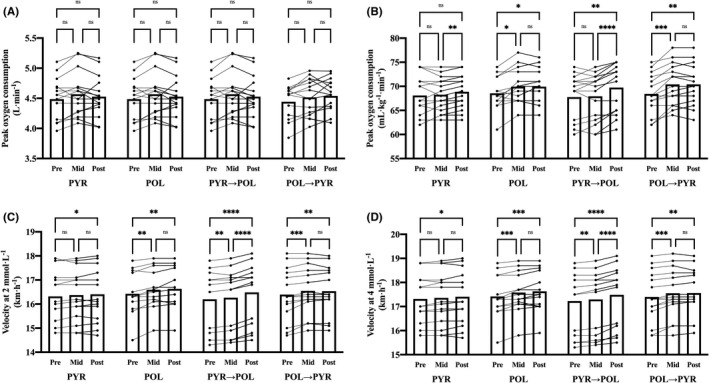
Changes between pre‐, mid‐ and post‐tests in the four different groups for absolute $\dot{V}O_{2\text{peak}}$ (A), relative $\dot{V}O_{2\text{peak}}$ (B), vBLa2 (C) vBLa4 (D). Significant difference between the tests (*p < 0.05; **p < 0.01; ***p < 0.001; ****p < 0.0001). No significant difference between the tests (^ns^ *P* >0.05). Data are presented individually for each participant and as overall mean

For body mass, there was a significant main effect of time (*F*(1.6, 91.0) = 6.4; *p* = 0.0046; $\eta_{p}^{2}$ = 0.10), while no interaction group × time was found (*F*(6, 112) = 0.9; *p* = 0.4946; $\eta_{p}^{2}$ = 0.05). For the absolute $\dot{V}O_{2\text{peak}}$ (Figure 2A) there was a significant main effect of time (*F*(1.9, 109) = 11.6; *p* < 0.0001; $\eta_{p}^{2}$ = 0.26), while no interaction group × time was found (*F*(6, 112) = 0.5; *p* = 0.8128; $\eta_{p}^{2}$ = 0.02). For the relative $\dot{V}O_{2\text{peak}}$ (Figure 2B), there was a significant main effect of time (*F*(1.4, 75.4) = 35.8; *p* < 0.0001; $\eta_{p}^{2}$ = 0.40) and an interaction group × time (*F*(6, 112) = 4.5; *p* = 0.0004; $\eta_{p}^{2}$ = 0.19). For vBLa2 (Figure 2C), there was a significant main effect of time (*F*(1.7, 93.2) = 62.6; *p* < 0.0001; $\eta_{p}^{2}$ = 0.53) and an interaction group × time (*F*(6, 112) = 6.8; *p* < 0.0001; $\eta_{p}^{2}$ = 0.27). For vBLa4 (Figure 2D), there was a significant main effect of time (*F*(1.6, 91.3) = 75.1; *p* < 0.0001; $\eta_{p}^{2}$ = 0.57) and an interaction group × time (*F*(6, 112) = 5.7; *p* < 0.0001; $\eta_{p}^{2}$ = 0.23). Percentage changes and effect sizes of pairwise comparisons pre‐ to mid‐tests, mid‐ to post‐tests and pre‐ to post‐tests in the four different groups are presented in the Table 5.

**TABLE 5:** Percentage changes and effect sizes of pairwise comparisons pre‐ to mid‐tests, mid‐ to post‐tests and pre‐ to post‐tests in the four different groups. Data are presented as mean ± standard deviation (Cohen's *d* effect size)

	PYR			POL			PYR → POL			POL → PYR		
	∆% (post‐pre)	∆% (mid‐pre)	∆% (post‐mid)	∆% (post‐pre)	∆% (mid‐pre)	∆% (post‐mid)	∆% (post‐pre)	∆% (mid‐pre)	∆% (post‐mid)	∆% (post‐pre)	∆% (mid‐pre)	∆% (post‐mid)
Body mass	−1.1 ± 1.7 (0.19)	−0.3 ± 1.9 (0.06)	−0.6 ± 3.0 (0.12)	−1.0 ± 1.6 (0.21)	−0.2 ± 1.6 (0.04)	−0.8 ± 2.1 (0.16)	−0.7 ± 1.5 (0.14)	−0.9 ± 1.8 (0.17)	0.2 ± 1.9 (0.04)	−0.7 ± 1.7 (0.16)	−1.2 ± 1.7 (0.27)	0.6 ± 2.3 (0.11)
Relative $\dot{V}O_{2\text{peak}}$	1.3 ± 2.2 (0.21)	0.4 ± 2.1 (0.05)	0.9 ± 0.7 (0.17)	2.1 ± 3.0 (0.40)	2.1 ± 2.6 (0.41)	0.0 ± 1.3 (0.00)	3.0 ± 2.8 (0.40)	0.3 ± 2.0 (0.04)	2.7 ± 1.6 (0.37)	3.0 ± 2.7 (0.47)	2.9 ± 2.0 (0.47)	0.0 ± 1.2 (0.00)
vBLa2	0.6 ± 0.7 (0.10)	0.4 ± 0.7 (0.06)	0.2 ± 0.5 (0.04)	1.3 ± 1.3 (0.25)	1.0 ± 0.9 (0.19)	0.3 ± 0.8 (0.07)	1.7 ± 0.7 (0.22)	0.4 ± 0.4 (0.06)	1.3 ± 0.7 (0.17)	0.9 ± 0.9 (0.13)	1.0 ± 0.8 (0.15)	−0.1 ± 0.5 (0.02)
vBLa4	0.6 ± 0.6 (0.10)	0.3 ± 0.4 (0.05)	0.3 ± 0.5 (0.05)	1.2 ± 1.1 (0.25)	0.9 ± 0.8 (0.19)	0.3 ± 0.8 (0.07)	1.5 ± 0.7 (0.22)	0.4 ± 0.3 (0.06)	1.1 ± 0.7 (0.16)	0.9 ± 0.8 (0.15)	1.0 ± 0.7 (0.16)	0.0 ± 0.5 (0.01)
Time trial time	−0.6 ± 0.6 (0.11)	−0.3 ± 0.4 (0.06)	−0.3 ± 0.5 (0.05)	−1.1 ± 1.1 (0.24)	−0.9 ± 0.7 (0.19)	−0.3 ± 0.7 (0.06)	−1.5 ± 0.7 (0.28)	−0.4 ± 0.3 (0.07)	−1.1 ± 0.7 (0.21)	−0.9 ± 0.8 (0.16)	−0.9 ± 0.7 (0.16)	0.0 ± 0.5 (0.01)
${\text{La}}_{\text{peak}}^{‐}$	7.2 ± 14.3 (0.24)	4.2 ± 12.2 (0.14)	4.0 ± 17.5 (0.11)	5.7 ± 11.8 (0.27)	5.7 ± 9.4 (0.27)	0.2 ± 9.2 (0.00)	1.5 ± 11.0 (0.08)	2.8 ± 14.0 (0.11)	0.6 ± 18.1 (0.03)	3.7 ± 12.7 (0.18)	4.4 ± 12.9 (0.21)	0.4 ± 15.2 (0.03)
HR_peak_	−0.2 ± 1.1 (0.02)	0.0 ± 1.2 (0.01)	−0.1 ± 1.6 (0.02)	−0.5 ± 0.9 (0.07)	−0.4 ± 1.3 (0.05)	−0.1 ± 1.6 (0.02)	0.1 ± 1.1. (0.02)	0.4 ± 1.0 (0.07)	−0.3 ± 1.5 (0.05)	−0.1 ± 1.1 (0.03)	0.1 ± 1.3 (0.02)	−0.2 ± 2.0 (0.05)
RPE	0.5 ± 5.7 (0.08)	0.2 ± 5.7 (0.00)	0.5 ± 6.2 (0.09)	2.1 ± 7.6 (0.37)	4.3 ± 6.2 (0.82)	−2.0 ± 6.8 (0.43)	1.4 ± 8.0 (0.22)	0.3 ± 7.9 (0.00)	1.4 ± 7.6 (0.22)	1.3 ± 6.9 (0.23)	2.8 ± 7.0 (0.55)	−1.3 ± 5.5 (0.29)

Abbreviations: $\dot{V}O_{2\text{peak}}$, peak oxygen consumption; ${\text{La}}_{\text{peak}}^{‐}$, peak blood lactate; HR_peak_, heart rate peak; POL → PYR, polarized + pyramidal training intensity distribution; POL, polarized training intensity distribution; PYR → POL, pyramidal + polarized training intensity distribution; PYR, pyramidal training intensity distribution; vBLa2, velocity at 2 mmol·L^−1^ of blood lactate concentration; vBLa4, velocity at 4 mmol·L^−1^ of blood lactate concentration.

### 3.5 5‐km time trial

**FIGURE 3:**
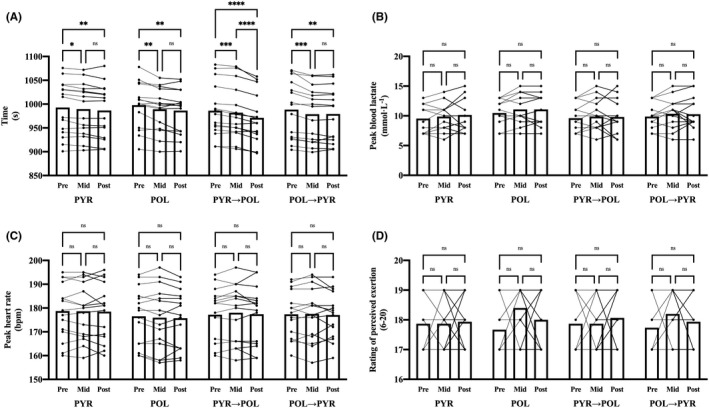
Changes between pre‐, mid‐ and post‐tests in the four different groups for time trial time (A), ${\text{La}}_{\text{peak}}^{‐}$ (B), HR_peak_ (C) and RPE (D). Significant difference between the tests (*p < 0.05; **p < 0.01; ***p < 0.001; ****p < 0.0001). No significant difference between the tests (^ns^ *p* >0.05). Data are presented individually for each participant and as overall mean

There was a significant main effect of time (*F*(1.7, 93.7) = 71.4; *p* < 0.0001; $\eta_{p}^{2}$ = 0.56) and an interaction group × time (*F*(6, 112) = 5.2; *p* = 0.0001; $\eta_{p}^{2}$ = 0.22) for the time trial performance (Figure 3A). There was a significant main effect of time (*F*(1.8, 99.1) = 4.5; *p* = 0.0164; $\eta_{p}^{2}$ = 0.08), while there was no interaction group × time (*F*(6, 112) = 0.3; *p* = 0.9578; $\eta_{p}^{2}$ = 0.02) for ${\text{La}}_{\text{peak}}^{‐}$ (Figure 3B). There were no main effects, nor interaction group × time (*F*(6, 112) = 0.5; *p* = 0.8208; $\eta_{p}^{2}$ = 0.03) for HR_peak_ (Figure 3C). There were no main effects, nor interaction group × time (*F*(6, 112) = 0.7; *p* = 0.6170; $\eta_{p}^{2}$ = 0.04) for RPE (Figure 3D). Percentage changes and effect sizes of pairwise comparisons pre‐ to mid‐tests, mid‐ to post‐tests and pre‐ to post‐tests in the four different groups are presented in the Table 5.

### 3.6 Variables that predicted time trial performance

Multiple regression analysis revealed that the variables that predict time trial performance at different timepoints (pre‐, mid‐ and post‐tests) are different compared to the ones that predict time trial performance enhancement from pre‐ to post‐tests (Table 6).

**TABLE 6:** Multiple regression models predicting time trial performance during pre‐, mid‐ and post‐tests, and performance enhancement

	Variables	Unstandardized coefficient *B*	Standardized coefficient *B*	SE	*t*	*p*
Pre‐tests						
Time trial performance (s)	(Constant)	1821.0	0.00	92.9	19.6	<0.0001
*F*(5, 54) = 166.8	Body mass	−1.2	0.05	0.8	1.4	0.1663
*p* < 0.0001	Relative $\dot{V}O_{2\text{peak}}$	69.0	5.19	28.3	2.4	0.0182
*R* ^2^ = 0.881	vBLa2	−86.9	−1.66	60.2	1.4	0.1545
	vBLa4	−233.3	−4.46	90.1	2.6	0.0124
	${\text{La}}_{\text{peak}}^{‐}$	0.8	0.03	1.2	0.6	0.5410
Mid‐tests						
Time trial performance (s)	(Constant)	1882.0	0.00	75.9	24.8	<0.0001
*F*(5, 54) = 66.4	Body mass	−0.9	−0.06	0.8	1.2	0.2295
*p* < 0.0001	Relative $\dot{V}O_{2\text{peak}}$	−1.7	−0.13	1.7	1.0	0.3191
*R* ^2^ = 0.861	vBLa2	34.2	0.66	54.7	0.6	0.5346
	vBLa4	−74.4	−1.45	52.8	1.4	0.1646
	${\text{La}}_{\text{peak}}^{‐}$	1.5	0.07	1.1	1.4	0.1661
Post‐tests						
Time trial performance (s)	(Constant)	1846.0	0.00	90.7	20.4	<0.0001
*F*(5, 54) = 65.6	Body mass	−1.1	−0.07	0.8	1.4	0.1607
*p* < 0.0001	Relative $\dot{V}O_{2\text{peak}}$	−2.2	−0.17	1.4	1.5	0.1395
*R* ^2^ = 0.859	vBLa2	−53.7	−1.04	60.0	0.9	0.3754
	vBLa4	13.7	0.27	60.0	0.2	0.8200
	${\text{La}}_{\text{peak}}^{‐}$	0.4	0.02	1.0	0.4	0.7175
Performance enhancement						
∆ Time trial performance (s)	(Constant)	−0.2	0.00	0.6	0.3	0.7370
*F*(5, 54) = 166.8	Body mass	−0.3	−0.04	0.3	1.2	0.2520
*p* < 0.0001	Relative $\dot{V}O_{2\text{peak}}$	−0.2	−0.04	0.2	1.3	0.1967
*R* ^2^ = 0.932	vBLa2	−10.7	−0.19	4.4	2.4	0.0183
	vBLa4	−45.3	−0.79	4.6	9.9	<0.0001
	${\text{La}}_{\text{peak}}^{‐}$	−0.1	−0.02	0.3	0.4	0.6559

Abbreviations: $\dot{V}O_{2\text{peak}}$, peak oxygen consumption; ${\text{La}}_{\text{peak}}^{‐}$, peak blood lactate; vBLa2, velocity at 2 mmol·L^−1^ of blood lactate concentration; vBLa4, velocity at 4 mmol·L^−1^ of blood lactate concentration.

During pre‐tests, time trial performance (in seconds) was predicted by the following equation:

1. time trial performance = –1.2 · body mass + 69 · relative $\dot{V}O_{2\text{peak}}$ – 86.9 · vBLa2 – 233.3 · vBLa4 + 0.8 · ${\text{La}}_{\text{peak}}^{‐}$ + 1821 (*R* ^2^ = 0.881, *p* < 0.0001).
2. During mid‐tests, time trial performance (in seconds) was predicted by the following equation:
3. time trial performance = –0.9 · body mass – 1.7 · relative $\dot{V}O_{2\text{peak}}$ + 34.2 · vBLa2 – 74.4 · vBLa4 + 1.5 · ${\text{La}}_{\text{peak}}^{‐}$ + 1882 (*R* ^2^ = 0.861, *p* < 0.0001).
4. During post‐tests, time trial performance (in seconds) was predicted by the following equation:
5. time trial performance = –1.1 · body mass – 2.2 · relative $\dot{V}O_{2\text{peak}}$ – 53.7 · vBLa2 + 13.7 · vBLa4 + 0.4 · ${\text{La}}_{\text{peak}}^{‐}$ + 1846 (*R* ^2^ = 0.859, *p* < 0.0001).
6. Time trial performance enhancement (in seconds) from pre‐ to post‐tests was predicted by the following equation:
7. ∆ time trial performance = –0.3 · ∆ body mass – 0.2 · ∆ relative $\dot{V}O_{2\text{peak}}$ – 10.7 · ∆ vBLa2 – 45.3 · ∆ vBLa4 – 0.1 · ∆ ${\text{La}}_{\text{peak}}^{‐}$ – 0.2 (*R* ^2^ = 0.939, *p* < 0.0001).
8. Body mass is expressed in kg, relative $\dot{V}O_{2\text{peak}}$ in ml·kg^−1^·min^−1^, vBLa2 and vBLa4 in km·h^−1^, and ${\text{La}}_{\text{peak}}^{‐}$ in mmol·L^−1^.

## 4 DISCUSSION

The main finding of this study was that a modification of training intensity distribution throughout a 16‐week periodization appeared to be slightly effective in improving performance in well‐trained runners. Changing the type of periodization from pyramidal to polarized in the second half of the periodization induced bigger improvements compared to the simple pyramidal and polarized ones, or compared to switching from polarized into pyramidal periodization.

The PYR → POL group's improvement was about 0.5% higher than in the other groups, both in the 5‐km time trial, in vBLa2 and vBLa4. In general, gains in time trial performance from pre‐ to post‐tests were between 0.6 and 1.7%, with the PYR → POL having at least a 5‐s further improvement compared to the other groups. Interestingly, this is similar to the typical error of high‐level athletes in completing middle‐ and long‐distance time trials.^28^ It follows that this threshold can be considered worthwhile for high‐level performing athletes.

These improvements in performance may appear modest, but a similar percentage gain in elite sports would have meant winning the heat or being excluded from the final in 75% of middle‐ and long‐distance events of athletics at the Tokyo 2020 Olympics. It must also be recognized that most of the significant changes reported in this study are below the smallest detectable change (SDC). Therefore, these changes are within the magnitude of the technical error and possibly do not represent true changes with statistical certainty. This is common in most intervention studies on high‐level athletes in sports science,^29^, ^30^ suggesting that relevant changes in the performance of athletes who are already close to their physiological and performance limits are very difficult to achieve through a single intervention.

### 4.1 5‐km time trial and lactate profiles

This study shows for the first time that the “pyramidal → polarized” periodization pattern seems to be more effective than the others in improving 5‐km time trial performance (even though below the SDC) in well‐trained runners. This is in line with the traditional approach of elite endurance athletes, where TID changes from an emphasis on a high‐volume, low‐intensity TID during the base period, toward a pyramidal TID during the pre‐competition phase, and finally to a polarized TID during the competitive phase.^7^

As $\dot{V}O_{2\text{peak}}$ change did not have a notable increase considering both the SDC and the discrepancies between absolute and relative values, the higher improvement in performance in the PYR → POL group could be a consequence of the higher improvement in vBLa2 and vBLa4, compared to other groups. Therefore, it is likely that improvement in running performance is mainly attributable to an enhanced running economy at these specific intensities. Indeed, as recently pointed out by Jones and colleagues,^31^ one of the limiting factors in endurance running performance is running economy. This factor becomes increasingly important as the athletes’ level rises, thus discriminating between athletes with similar $\dot{V}O_{2\text{peak}}$ (as in the present study). All this, together with the results of the multiple linear regression where we showed that performance enhancements were mainly attributable to improvements in vBLa2 and vBLa4, further demonstrates that submaximal variables (eg, vBLa2 and vBLa4) have a significantly greater correlation with performance improvements (and, more generally, with performance) than maximal variables (eg, $\dot{V}O_{2\text{peak}}$, ${\text{La}}_{\text{peak}}^{‐}$, etc.). However, we must recognize that vBLa2 and vBLa4 include running, whereas $\dot{V}O_{2\text{peak}}$, ${\text{La}}_{\text{peak}}^{‐}$ where just physiological measures and do not integrate any performance measure. Measuring velocity associated with these physiological variables could have potentially led to different results.

### 4.2 $\dot{V}O_{2\text{peak}}$

$\dot{V}O_{2\text{peak}}$ results are in line with what was assumed about the relative importance of the maximal variables in high‐level performance in homogeneous groups. Significant changes of relative VO_2peak_ (even below the SDC) were found in all the three groups with different effect sizes, while no effects were found on absolute VO_2peak_. The highlighted trend of increase in relative VO_2peak_ in the different groups is mainly attributed to weight variations and not to real changes in absolute VO_2peak_.

The different results between the 5‐km time trial performance, lactate profiles and the absolute/relative $\dot{V}O_{2\text{peak}}$ are consistent with the critical discriminative role of $\dot{V}O_{2\text{peak}}$. In fact, $\dot{V}O_{2\text{peak}}$ helps to discern among different categories of athletes and highlights general improvements in aerobic fitness. However, it is not the main determinant factor in homogeneous groups of athletes.^32^ Indeed, a higher $\dot{V}O_{2\text{peak}}$ is not always associated with superior endurance running performances. Physiological threshold and running economy have been demonstrated to be better predictors.^33^ Recent studies have in fact shown that high‐level elite runners might have similar $\dot{V}O_{2\text{peak}}$ compared to lower‐level elite athletes.^33^

### 4.3 Mechanistic explanation

A clear mechanism that explains why the “pyramidal → polarized” periodization pattern leads to superior physiological and performance improvements remains undefined. One of the most likely explanations could lie in the role of training intensity in the peaking process.^34^ On one hand, it has been demonstrated that peak performance is achieved by increasing relative intensity and decreasing volume of training during the tapering phase.^35^ On the other hand, certain guidelines of this phenomenon have been proposed^35^ but are rarely followed by well‐trained or elite athletes in their yearly plans.^7^, ^11^, ^36^

Traditionally, training intensity is considered of paramount importance to maximize the physiological and performance adaptations of well‐trained athletes, showing its greatest role in the consolidation of these benefits in the 14 days prior to the athletes’ main target race.^11^ Thus, peaking cannot be explained by a single value; rather, it is the result of a combination of muscular, cardiovascular, hormonal and psychological factors derived from high intensity training^37^, ^38^ which act in synergy to maximize training adaptations. According to this logic, a gradual increase of intensity throughout a training program can facilitate the achievement of peak performance in correspondence to the goal. We have shown how an early increase in intensity in the first 8 weeks, as occurred in the POL group, leads to a relevant improvement in performance only in the mid‐tests, while remaining almost unchanged after the second 8 weeks.

One of the strengths of the present study is that training load was constant between all four groups to allow for isolated manipulations of TID. This approach can provide a unique insight into the effects of periodization patterns on performance outcomes in high‐level athletes, where it is often difficult to control for these types of interventions. In parallel, if the explanation behind our results lies mainly in the peaking effect of intensity, we could hypothesize that a reduction in training volume and load in the last 2–3 weeks before post‐tests would have further amplified the results, as traditionally occurs with pre‐competition tapering.^35^

### 4.4 Limitations

The present study has some limitations that warrant a brief discussion. First, we acknowledge the limitations of using HR/TRIMPS for training quantification. It is a fact that, for different reasons (eg, overreaching, dehydration, etc.), HR can respond unexpectedly during training sessions for a similar external training load. In fact, a lower systematic response during training could be expected when compared to the values recorded during incremental tests. Second, even though the polarization index provides an objective cut‐off to distinguish polarized from non‐polarized distributions, it does not allow differentiation between sub‐types of the non‐polarized TID structures. For this reason, we decided to use this method just as a confirmation of the nature of the TID. Third, the 5‐km run is mainly dependent upon the aerobic energy system, so other outputs may be expected in other competitive distances where other metabolic components are predominant.

## 5 PERSPECTIVE

Endurance runners seem to benefit from a change in the final phases of the periodization, from a pyramidal to a polarized model. This increase in relative intensity could favor the pre‐competition peaking phase and maximize performance improvements. More generally, this study showed how periodization based on high volumes in Z1 and reduced volumes in Z2 and Z3 allows for significant improvements even for well‐trained runners, confirming that these types of distributions are the most effective for endurance athletes. Future studies are needed to confirm that the same findings could be applied to runners of a lower or higher performance level. It would also be interesting to check if the results could be extended to other endurance disciplines (ie, cycling, cross‐country skiing, etc.), where mechanic loads are less strenuous than running, allowing for more total volume per week. Moreover, it would be relevant to verify whether a training program with a higher percentage of accumulated training time at higher training intensities would be even better, and if there is a threshold training intensity in Z1 below which no physiological adaptation really occurs. Finally, future research should aim to understand the physiological foundations behind these findings.

In conclusion, a 16‐week training periodization seems to be effective in improving performance, albeit not physiological ones, of well‐trained endurance runners. Switching from pyramidal into polarized after 8 weeks of periodization appeared to be more efficacious in maximizing performance improvements, compared to the other forms of periodization (pyramidal, polarized and polarized followed by pyramidal).

## CONFLICT OF INTEREST

Authors declare no conflict of interests.

## Data Availability

The data that support the findings of this study are available from the corresponding author upon reasonable request.
